# Proanthocyanidins, from *Ribes nigrum* leaves, reduce endothelial adhesion molecules ICAM-1 and VCAM-1

**DOI:** 10.1186/1476-9255-2-9

**Published:** 2005-08-09

**Authors:** N Garbacki, M Kinet, B Nusgens, D Desmecht, J Damas

**Affiliations:** 1 Laboratoire de Physiologie humaine, CHU, Tour 3, Université de Liège, Avenue de l'Hôpital, 3, B-4000 Sart Tilman, Belgium; 2 Laboratoire de Biologie des Tissus Conjonctifs, Tour 3, Université de Liège, Avenue de l'Hôpital, 3, B-4000 Sart Tilman, Belgium; 3 Département de Morphologie et de Pathologie, Pathologie générale, Faculté de Médecine vétérinaire, Université de Liège, bvd de Colonster, 20, B-4000 Sart Tilman, Belgium

## Abstract

**Background:**

The effects of proanthocyanidins (PACs), isolated from blackcurrant (*Ribes nigrum *L.) leaves, on neutrophil accumulation during inflammatory processes were investigated *in vivo *and *in vitro*.

**Methods:**

*In vivo *studies were performed using carrageenin-induced pleurisy in rats pre-treated with PACs. Exudate volume and PMNs accumulation were measured. Leukocyte cell adhesion molecules (LFA-1, Mac-1 and VLA-4) mobilization in circulating granulocytes were analysed by flow cytometry and endothelial cell adhesion molecules (ICAM-1 and VCAM-1) were detected by immunohistochemistry on lung sections.

*In vitro *studies were conducted on endothelial LT2 cells, stimulated with TNF-α, to evaluate ICAM-1, IL-8 and VEGF mRNA expression upon PACs treatment.

Data sets were examined by one-way analysis of variance (ANOVA) followed by a Scheffe post-hoc test.

**Results:**

Pretreatment of the animals with PACs (10, 30 and 60 mg/kg) inhibited dose-dependently carrageenin-induced pleurisy in rats by reducing pleural exudate formation and PMNs infliltration. Leukocyte cell adhesion molecules mobilization was not down-regulated on granulocytes by PACs. Immunohistochemistry on lung sections showed a decreased production of endothelial cell adhesion molecules.

*In vitro *experiments demonstrated that PACs were able to significantly inhibit ICAM-1 but not IL-8 and VEGF_165 _mRNA expression. Moreover, VEGF_121 _mRNA expression was dose-dependently enhanced.

**Conclusion:**

This study provides evidence to support the anti-inflammatory activity of proanthocyanidins is related to an inhibition of leukocyte infiltration which can be explained at least in part by a down-regulation of endothelial adhesion molecules, ICAM-1 and VCAM-1 and that these compounds are capable of modulating TNF-α-induced VEGF transcription.

## Introduction

Cell-cell and cell-extracellular matrix interactions play a central role in cell migration and leukocyte activation during the inflammatory process. Leukocyte adhesion to the endothelial cells and recruitment to extravascular tissue are fundamental events in the pathogenesis of the inflammatory disease. All the steps in the recruitment cascade are orchestrated by cell-adhesion molecules (CAMs) expressed on both leukocytes and endothelial cells, and different subsets of CAMs are responsible for the different steps in leukocyte extravasation [1]. This cascade includes the selectins, the integrins and members of the immunoglobulin superfamily. The expression of these adhesion molecules is induced by various stimuli including cytokines such as TNF-α, IL-1β, chemokines such as IL-8, lipid mediators and peptides [2].

ICAM-1 (Intercellular Adhesion Molecule-1, also designated as CD54) and VCAM-1 (Vascular Cell Adhesion Molecule-1, also designated as CD106) are members of immunoglobulin superfamily involved in cell adhesion. ICAM-1 is normally found on the surface of endothelial cells, but its expression can be significantly increased upon endothelial cell activation with cytokines or endotoxin. ICAM-1 binds to integrin members of the beta-1 subfamily such as LFA-1 (Lymphocyte Function-associated Antigen-1, also designated as CD11a/CD18) and Mac-1 (Macrophage antigen-1, also designated as CD11b/CD18). Leukocyte adhesion to ICAM-1 is performed by LFA-1 upregulation and conformational change and by Mac -1 mobilisation to the cell surface by fusion of storage granules to the cell membrane [3]. VCAM-1, a transmembrane endothelial protein, is the counter-receptor of VLA-4 (Very Late Antigen-4, also designated as α4β1 or CD49d/CD29). This latter beta-1 integrin mediates the adhesion of lymphocytes, monocytes, eosinophils and natural killer cells to activated endothelial cells [4].

VEGF (Vascular Endothelial Growth Factor) and IL-8 are two major mediators involved in inflammatory processes associated with angiogenesis [5,6]. VEGF functions as an endothelial growth and survival factor, it also increases endothelial permeability [5] and is particularly relevant to neutrophils emigration into the lung [7]. It causes vasodilatation through the nitric oxide synthase pathway in endothelial cells [8] and can activate migration of neutrophils [7,9,10]. IL-8 stimulates the ability of neutrophils to invade injured tissues. Besides its chemotactic influence, IL-8 also stimulates a rapid Mac-1 as well as LFA-1 expression on neutrophils which enables the adherence of these cells to activated vascular endothelial cells [11].

Proanthocyanidins (PACs) are a group of biologically active polyphenols, synthesized by many plants. PACs isolated from blackcurrant (*Ribes nigrum *L.) leaves are monomers and oligomers of flavan-3-ol units, the prodelphinidins and the procyanidins [12,13]. These compounds have been reported to have anti-inflammatory properties *in vivo *and *in vitro *[14-17]. We have previously shown that PACs decrease the accumulation of circulating leukocytes and plasma exudation during carrageenin acute inflammatory reaction induced in rats. This anti-inflammatory effect was associated with a reduction of pro-inflammatory cytokines [18]. The current study was undertaken to build on that previous finding and investigate the effects of the PACs on adhesion molecules during inflammatory processes.

## Materials and methods

### Proanthocyanidins

Proanthocyanidins from *Ribes nigrum *leaves were extracted and isolated according to a previously described method [12]. A voucher sample (RN 210590) has been deposited in the Pharmaceutical Institute of Liège, Belgium. Briefly, leaves were powdered separately and then extracted at room temperature with acetone (70 % v/v in water). The acetone was removed under vacuum at 40°C. The resulting aqueous solution was freeze-dried. Isolation of monomers and oligomers was carried out by MPLC on reversed-phase RP8 with water-acetone (9:1) to obtain a total proanthocyanidin-enriched fraction (PACs).

### Carrageenin-induced pleurisy in rats

#### Animals

Mae Wistar rats, weighing 250 – 300 g were used. The animals were maintained on a standard laboratory diet with free access to water. The experiments were conducted as approved by the Animal Ethics Committee of the University of Liège, Belgium.

#### Carrageenin-induced pleurisy

Rats were pretreated with an intraperitoneal (i.p.) injection of saline or PACs (10, 30 or 60 mg/kg) 30 min before the intrapleural injection of carrageenin. They were then anaesthetized with ketamine HCl (75 mg/kg, i.p.) and carrageenin (0.2 ml, 10 mg/ml) or saline (0.2 ml) was administered into the right pleural cavity. Each experimental group contained 6 animals. Four hours later, the animals were anaesthetized with sodium pentobarbital (80 mg/kg, i.p.). The peritoneal cavity was opened and blood (3–5 ml) was withdrawn into a heparinized syringe from the inferior vena cava.

The chest was carefully opened and the pleural cavity rinsed with 2.0 ml saline solution containing heparin (5 U/ml). Exudates and washing solutions were removed by aspiration. Exudates with blood were rejected. The volume of the exudates was calculated by subtracting the volume of the washing solution (2.0 ml) from the total volume recovered. A sample of each exudate was diluted in phosphate buffer and polymorphonuclear leukocytes count was performed using a hemocytometer.

After removal of the exudates, lungs were withdrawn and fixed for one day under 30 cm pressure with 10% formaldehyde aqueous solution containing 0.480 M Na_2_HPO_4 _and 0.187 M KH_2_PO_4 _(pH 7.2) at room temperature. They were then dehydrated by graded ethanol and embedded in Paraplast.

#### Production of LFA-1, Mac-1 and VLA-4

The relative expression by circulating granulocytes of LFA-1, Mac-1 and VLA-4 were measured using flow cytometry. Granulocytes (200 000 cells) were stained by saturating concentrations of monoclonal antibodies: R-PE-conjugated WT.1 against rat CD11a (0.1 μg per million cells), FITC-conjugated WT.5 against CD11b (1 μg per million cells) or FITC-conjugated MRα4-1 against CD49d (0.1 μg per million cells).

After incubation of whole blood (40 μl), withdrawn into a heparinized syringe from the inferior vena cava, for 20 min in the dark at room temperature, erythrocytes were lysed with ammonium chloride buffer (30 min, in the dark at room temperature). The cells were finally washed three times with phosphate buffer saline containing 0.2% bovine serum albumin and 0.09% sodium azide (pH 7.4), fixed with 4% paraformaldehyde in PBS and then analyzed on a flow cytometer.

For this experiment, aspirin was used as a reference drug and was administrated intraperitoneally to rats at 200 mg/kg.

The antibodies were supplied by BD Pharmingen (Erembodegem, Belgium).

#### Immunohistochemical localization of ICAM-1 and VCAM-1

Indirect immunohistochemical staining was performed on 7 μm thick lung sections (2 sections per animal). After deparaffinization with UltraClear, endogenous peroxidase was quenched with 3% H_2_O_2 _in H_2_O for 10 min. The sections were permeabilized with 0.1% Tween 20 in PBS for 10 min and then incubated overnight with a 1/100 primary mouse anti-rat ICAM-1 monoclonal antibody or a 1/500 primary mouse anti-rat VCAM-1 monoclonal antibody or with control solution (buffer alone). Specific labelling was detected by incubating the sections 30 min at room temperature with a 1/100 biotin-conjugated goat anti-mouse IgG. Streptavidin-HRP complex was then added to cover the tissue sections and incubated 30 min at room temperature. DAB substrate was applied to the sections, incubated for 5 min and finally washed in water. Tissue sections were then counterstained with hematoxylin-eosin, dehydrated and examined using light microscopy.

The antibodies, streptavidin-HRP complex pre-diluted and DAB substrate kit were supplied by BD Pharmingen (Erembodegem, Belgium).

### ICAM-1, IL-8 and VEGF mRNA expression in LT2 endothelial cells

#### Cell culture

LT2 cell line originates from human umbilical vein endothelium cells (HUVEC) which have been immortalized by transfection with Large T SV40 antigen (a kind gift of E. Dejana, Milan, to one of us BN). Cells were grown at 37°C in a humidified 95% air-5% CO_2 _atmosphere, using 2% pork skin gelatin-coated petri dish and RPMI 1640 medium supplemented with 10% FCS, 45 U/ml penicillin and 45 μg/ml streptomycin.

Cytotoxicity of PACs on LT2 cells was previously tested using the MTT assay (Sigma, Bornem, Belgium), based on conversion by mitochondrial dehydrogenases of the substrate (MTT) containing tetrazolium ring into the blue formazan detectable spectrophotometrically [19]. The level of blue formazan is then used as indirect index of cell density. The optical density of each sample was measured with a microplate spectrophotometer reader at 560 nm. Three replicates were used for each sample.

#### mRNA determination using RT-PCR

LT2 cells (100 000 cells), pretreated 24 h with PACs (10, 30 and 60 μg/ml) or with PBS (controls), were activated in triplicate with TNF-α (5 ng/ml, 12 h). The medium was then discarded and the cells frozen (-70°C). The level of mRNA was determined using RT-PCR as described below.

#### RNA extraction

The total RNA was isolated using High Pure RNA Isolation kit (Roche Applied Science, Penzberg, Germany). Briefly, endothelial cells were treated by a lysis buffer which contains guanidine hydrochloride. The lysates were laid on filter tubes, where RNA was bound, and contaminating DNA eliminated with DNase containing buffer. The bound RNA was purified from salts, proteins and other cellular impurities by washing steps and finally eluted in water. Quantification of RNA was assayed by Ribo Green RNA Quantification kit (Molecular Probes Europe BV, Leiden, The Netherlands) and RNA samples were diluted to 4 ng/μl.

#### One step RT-PCR

The one-step RT-PCR reactions were performed in duplicate using the GeneAmp Thermostable rTth Reverse Transcriptase RNA PCR kit (Applied Biosystems, Roche Molecular Systems, New Jersey, USA), specific pairs of primers (Table 1), 10 ng of total RNA and, when available, a known copy number of an original synthetic internal standard of RNA, in 25 μl reaction mixture [20].

**Table 1:** Specific pairs of primers used for RT-PCR analysis.

	Reverse (5'-3')	Forward (5'-3')
28S	-GATTCTGACTTAGAGGCGTTCAGT-	-GTTCACCCACTAATAGGGAACGTGA-
ICAM-1	-TCCAGTTCAGTGCGGCACGAGAA-	-CTGATGGGCAGTCAACAGCTAAAA-
IL-8	-GAATTCTCAGCCCTCTTCAAAAAC-	-GCCAAGGAGTGCTAAAGAACTTAG-
VEGF	-CTCACCGCCTCGGCTTGTCACA-	-CCTGGTGGACATCTTCCAGGAGTA-

The RT step (70°C, 15 min) was followed, after an initial denaturation for 2 min at 95°C, by PCR amplification for the adequate number of cycles and terminated by a final elongation step of 2 min at 72°C. The PCR conditions for amplification of ICAM-1 (30 cycles) and the 28S RNA (17 cycles) were: 94°C for 15 sec; 66°C for 20 sec; 72°C for 10 sec. For IL-8 (25 cycles), the conditions were: 94°C for 15 sec, 60°C for 30 sec, 72°C for 10 sec. For VEGF (32 cycles), the conditions were: 94°C for 20 sec, 66°C for 30 sec, 72°C for 1 min.

A 10 μl aliquot from each PCR reaction were subjected to electrophoresis in 10% polyacrylamide gel and analysed using a Fluor-S-MultiImager (BioRad, Hercules, Ca) after staining with GelStar (FMC BioProducts, Rockland, ME, USA) dye. The results were expressed in arbitrary units per unit of 28S RNA. The 28S, ICAM-1 and VEGF primers were commercially synthesized by Invitrogen (Merelbeke, Belgium) and IL-8 primers by Eurogentec (Liège, Belgium).

### Statistical evaluation

Results are given as mean ± standard error of the mean (s.e. mean) of N observations. Data sets were examined by one-way analysis of variance (ANOVA) followed by a Scheffe post-hoc test. A P-value of less than 0.05 was considered significant.

## Results

### Carrageenin-induced pleurisy

**Figure 1:**
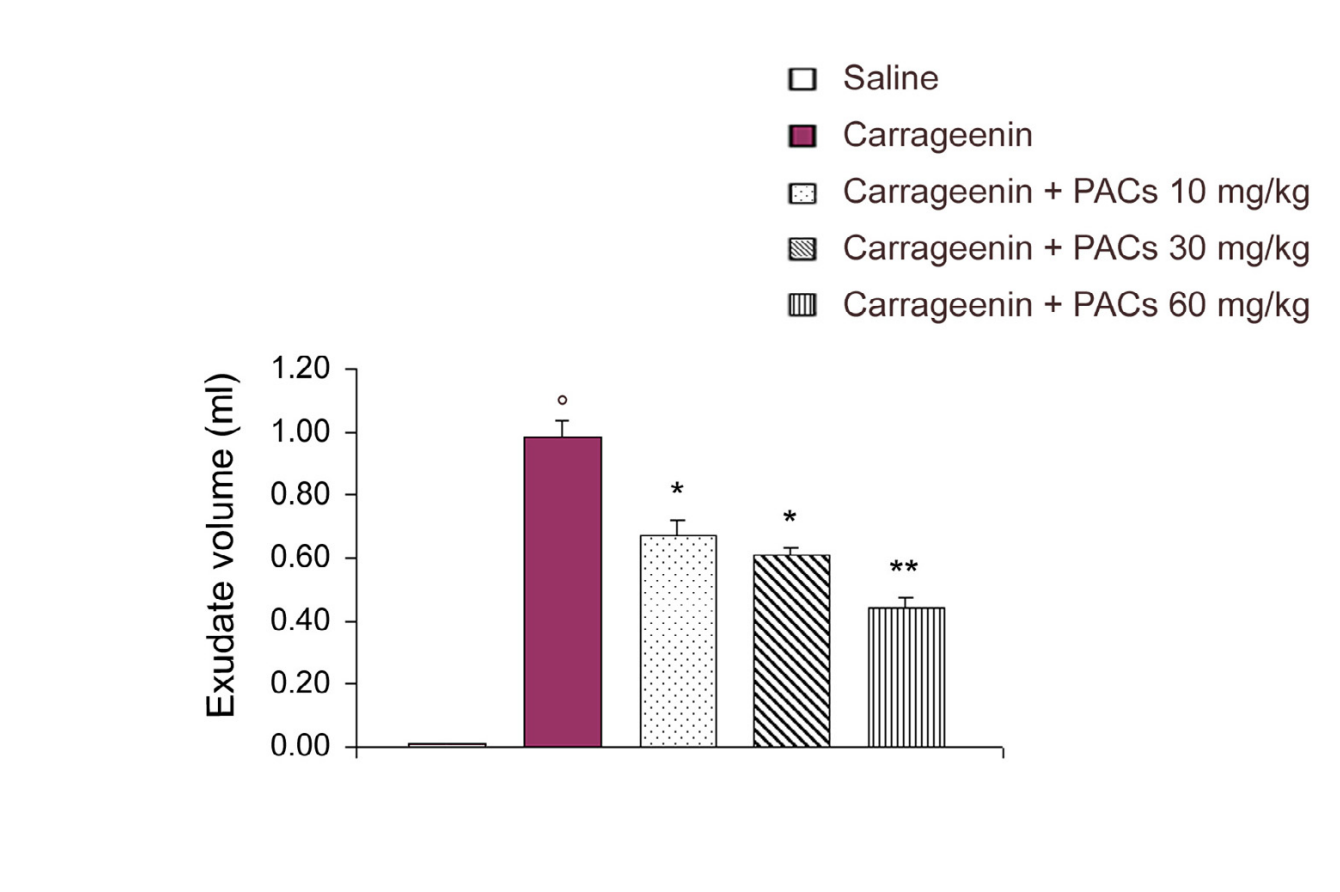
**Effect of PACs on exudate volume during carrageenin-induced pleurisy. **At 4 h after intrapleural injection of carrageenin, the volume of the exudate was reduced by PACs (10, 30 and 60 mg/kg) administration. Each value is the mean ± s.e. mean of n = 6 experiments. °P < 0.01 versus saline. *P < 0.05 versus carrageenin. **P < 0.01 versus carrageenin.

**Figure 2:**
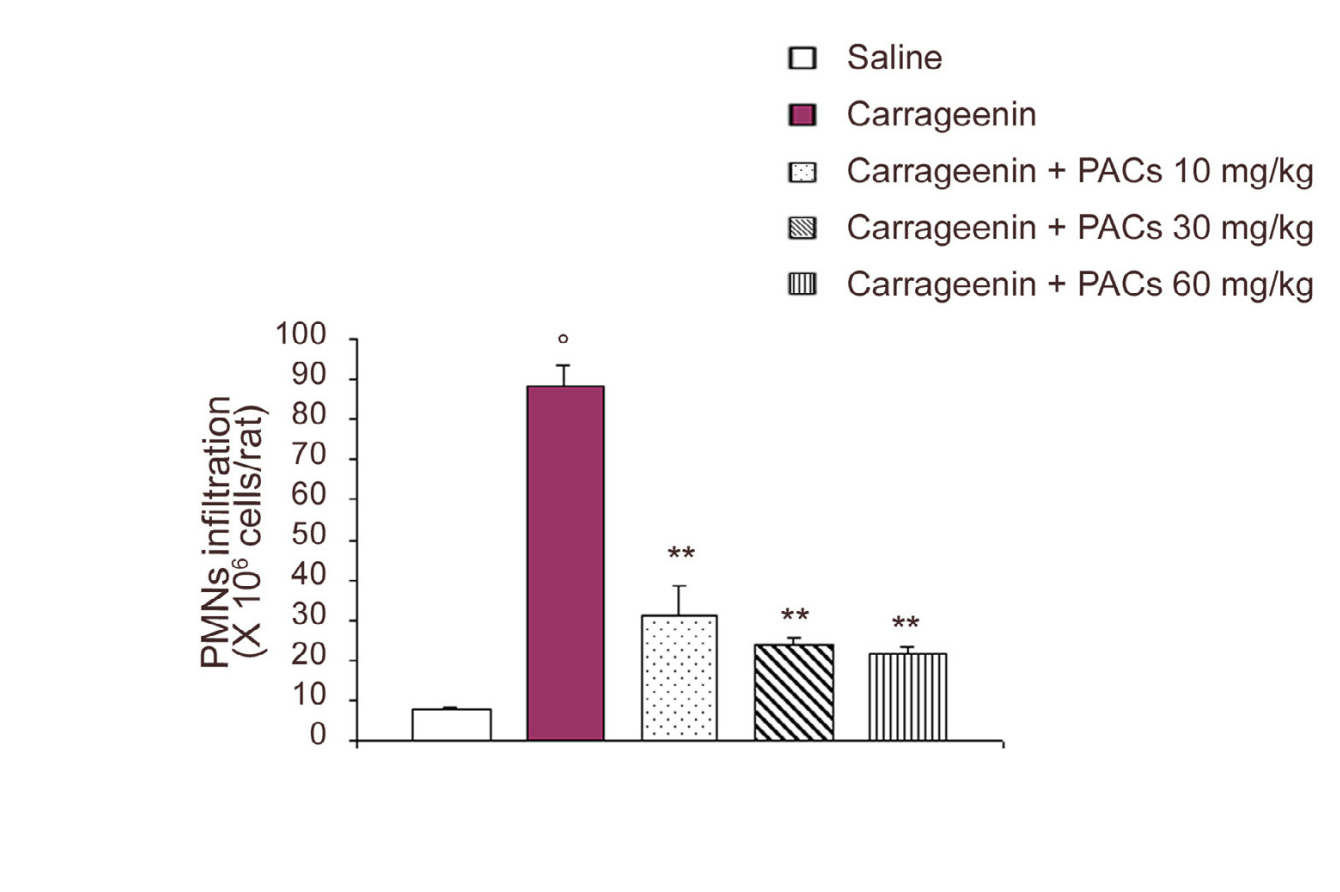
**Effect of PACs on PMNs infiltration during carrageenin-induced pleurisy. **At 4 h after intrapleural injection of carrageenin, the accumulation of PMNs in the pleural cavity was dramatically inhibited by PACs (10, 30 and 60 mg/kg). Each value is the mean ± s.e. mean of n = 6 experiments. °P < 0.01 versus saline. **P < 0.01 versus carrageenin.

In control rats, the volume of the exudate collected 4 h after carrageenin injection reached 0.98 ± 0.06 ml per rat (n = 6) (Figure 1). This exudate contained a large number of cells, mostly (> 95%) polymorphonuclear leukocytes (PMNs). The total leukocyte number in the exudate was 88.53 ± 5.4 × 10^6 ^per rat (Figure 2). PACs (10, 30 and 60 mg/kg) significantly reduced the volume of the exudate in a dose-dependent relationship (by, respectively, 31, 37 and 55%). Moreover, PMNs infiltration was also significantly inhibited by PACs in a dose-dependent way (by, respectively, 64, 73 and 75%).

### Production of LFA-1, Mac-1 and VLA-4

In order to examine whether PACs affect the expression of adhesion molecules on leukocytes during carrageenin-induced pleurisy, the expression of three different molecules: LFA-1 (CD11a/CD18), Mac-1 (CD11b/CD18) and VLA-4 (CD49d/CD29) was measured by flow cytometry.

**Figure 3:**
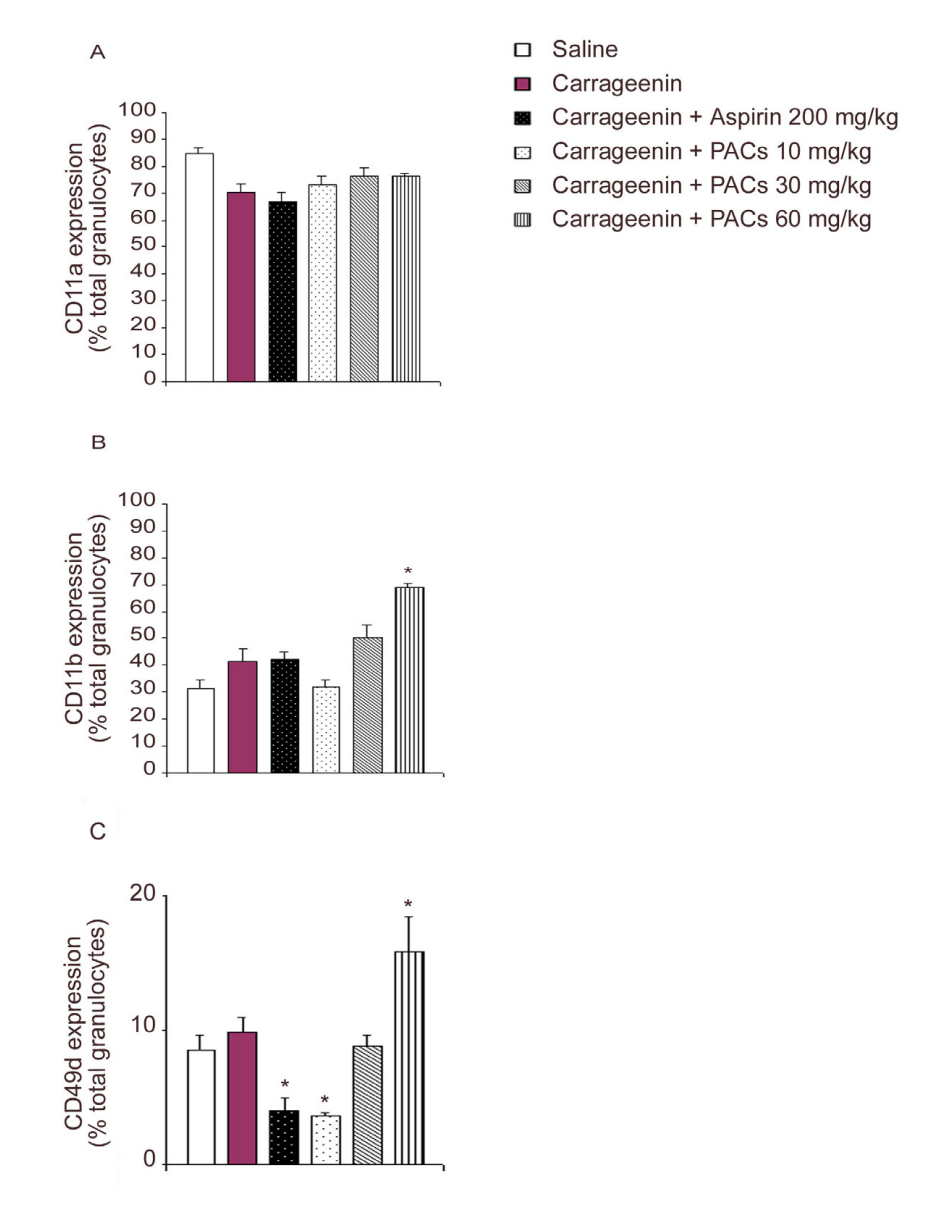
**Effect of PACs and aspirin on leukocyte cell adhesion molecules during carrageenin-induced pleurisy. **The relative expression by circulating granulocytes of LFA-1, Mac-1 and VLA-4 were measured using flow cytometry. Granulocytes were incubated with saturating concentrations of monoclonal antibodies (mAb): R-PE-conjugated WT.1 against rat CD11a (LFA-1) (A), FITC-conjugated WT.5 against CD11b (Mac-1) (B) or FITC-conjugated MRα4-1 against CD49d (VLA-4) (C). mAb against CD11a gave negative results for all tested groups. PACs induced up-regulation of CD11b and CD49d with the highest dose tested and down-regulation of CD49d with the lowest dose. Aspirin also down-regulated CD49d at 200 mg/kg. Each value is the mean ± s.e. mean of n = 6 experiments. *P < 0.05 versus carrageenin.

None of these molecules was significantly modulated during carrageenin-induced pleurisy (Figure 3). Aspirin reduced the level of VLA-4, as also did PACs at 10 mg/kg (Figure 3C) but at 60 mg/kg PACs increased the expression of Mac-1 (Figure 3B) and VLA-4 (Figure 3C).

### Immunohistochemical localization of ICAM-1 and VCAM-1

**Figure 4:**
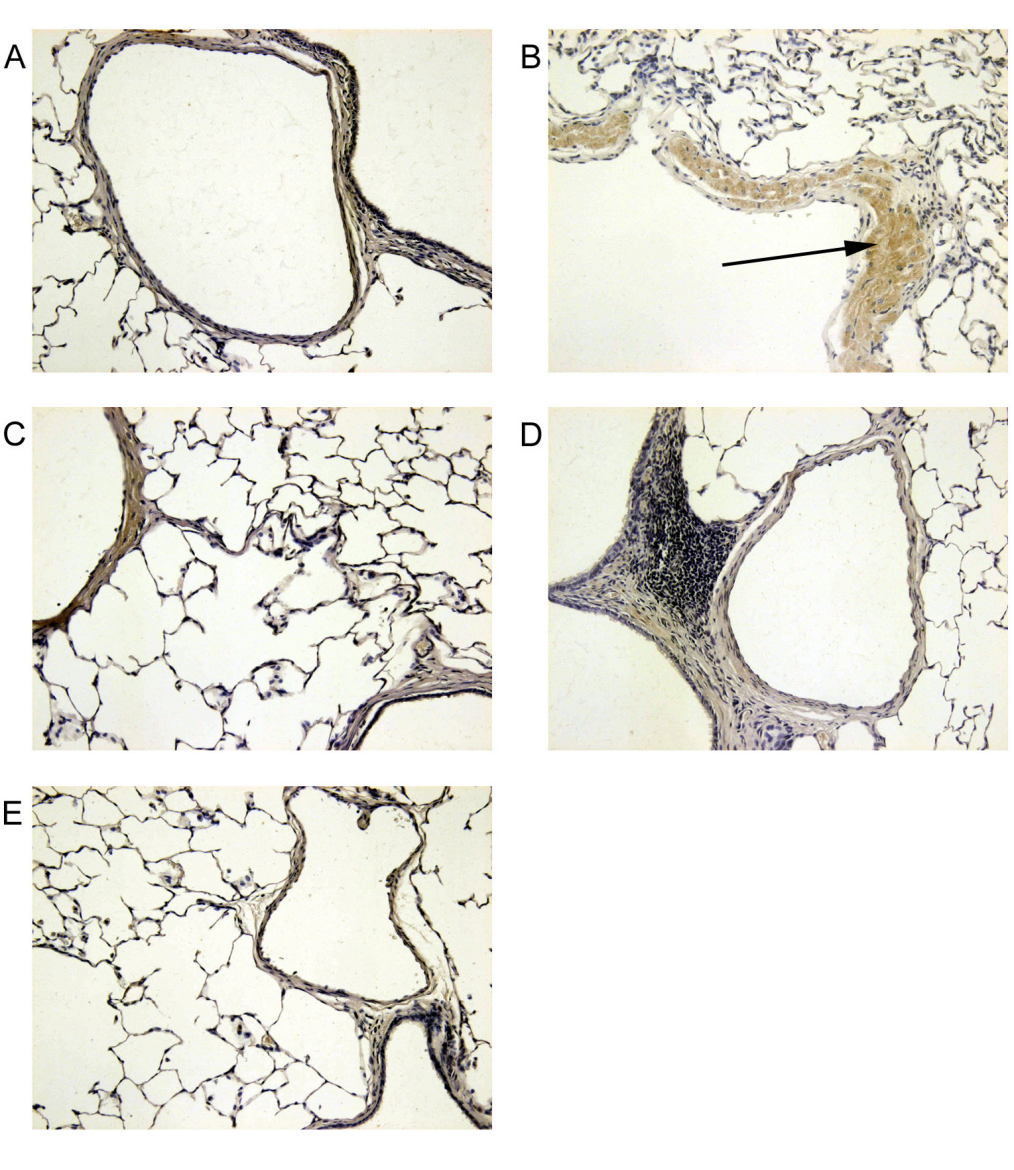
**Immunohistochemical localization of ICAM-1 in the lung. **When compared to lung sections taken from sham group (A), lung sections from carrageenin-treated rats (B) demonstrated intense positive brown staining for ICAM-1 (black arrow). The degree of tissue staining for ICAM-1 from carrageenin-treated rats that had received PACs (10, 30 and 60 mg/kg, respectively C, D and E) was markedly reduced. Original magnification: × 125. This figure is representative of six experiments performed on different animals.

**Figure 5:**
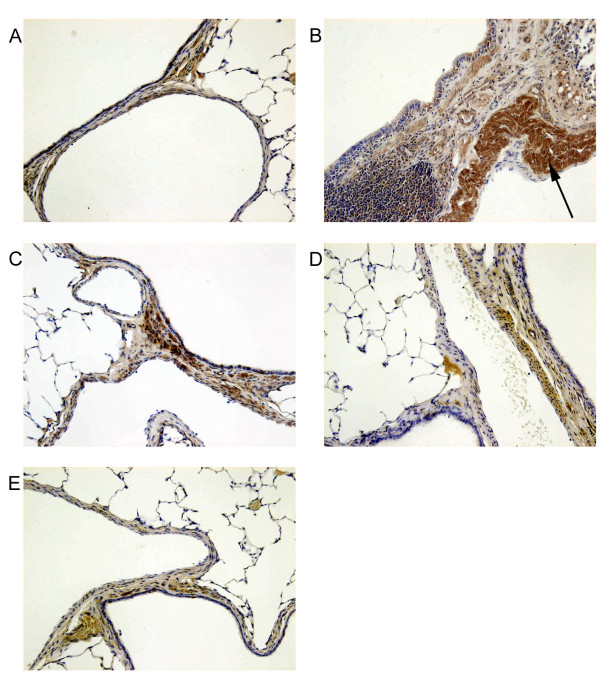
**Immunohistochemical localization of VCAM-1 in the lung. **When compared to lung sections taken from sham group (A), lung sections from carrageenin-treated rats (B) demonstrated intense positive brown staining for VCAM-1 (black arrow). The degree of tissue staining for VCAM-1 from carrageenin-treated rats that had received PACs (10, 30 and 60 mg/kg, respectively C, D and E) was markedly reduced. Original magnification: × 125. This figure is representative of six experiments performed on different animals.

Immunohistochemical analysis of lung sections obtained from animals, treated with carrageenin alone, revealed positive staining for ICAM-1 (Figure 4) and VCAM-1 (Figure 5). In contrast, sham group showed poor staining. In lung tissue obtained from PACs pre-treated rats, immunohistochemical staining for ICAM-1 and VCAM-1 was lower than those of positive control and disappeared with the highest doses tested to reach negative control level at 60 mg/kg.

### ICAM-1, VEGF and IL-8 mRNA expression

**Figure 6:**
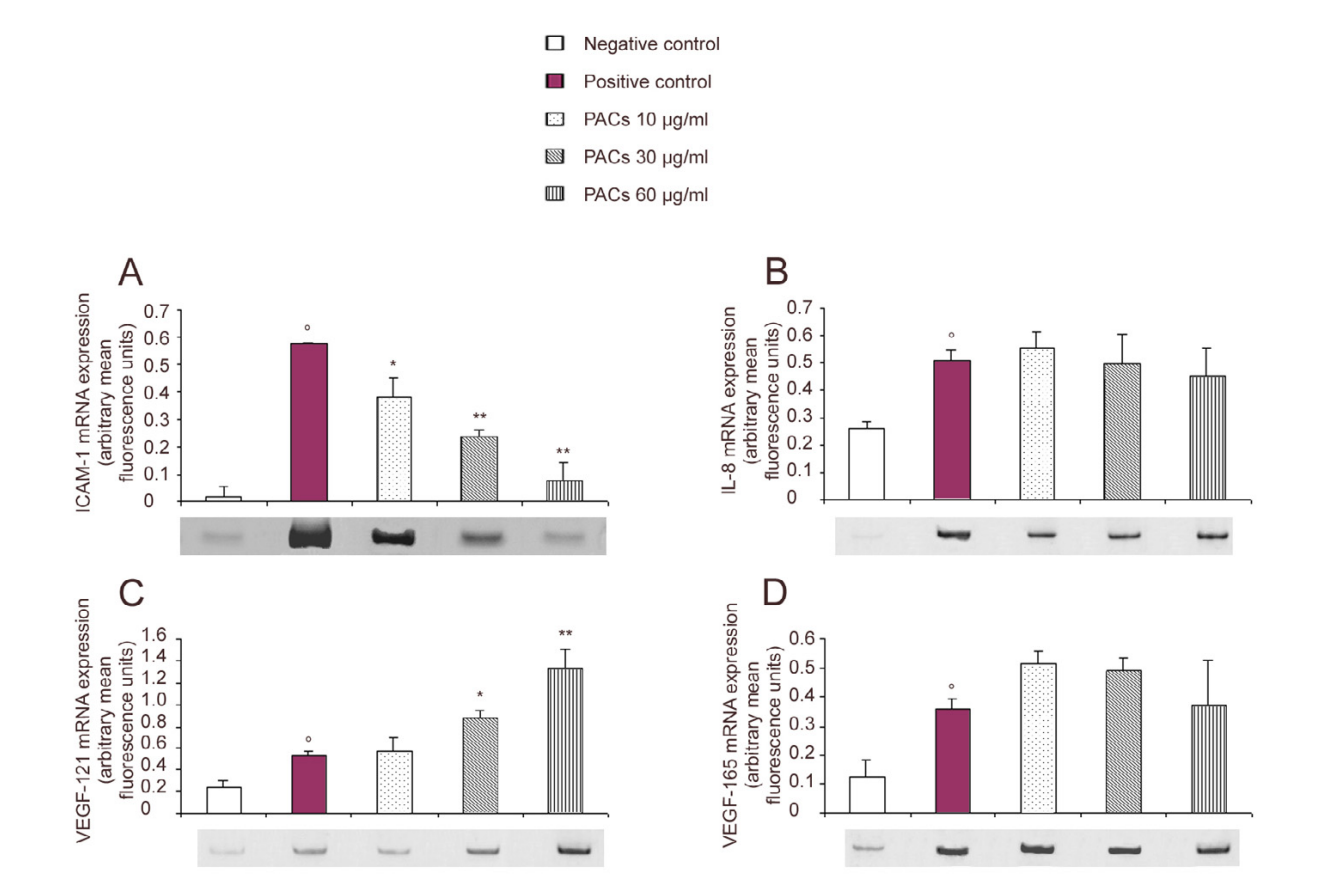
**Effect of PACs on TNF-α-induced ICAM-1, IL-8, VEGF_121 _and VEGF_165 _mRNA expression in human endothelial LT2 cells. **LT2 cells were pretreated with PACs (10, 30 and 60 μg/ml) or PBS for 24 h and then activated with 5 ng/ml TNF-α for 12 h. PACs dose-dependently inhibited mRNA expression of ICAM-1 (A), maintained IL-8 (B) and VEGF_165 _(D) and up-regulated dose-dependently VEGF_121 _mRNA expression (C). Gel electrophoresis of PCR products is shown under X-axis. Each value is the mean ± s.e. mean of three experiments analysed in duplicate each. °P < 0.01 versus negative control. *P < 0.05 versus positive control. **P < 0.01 versus positive control.

PACs were tested from 5 to 100 μg/ml on LT2 cells during 24 h and were not found to be significantly cytotoxic for all the used doses. The effect of PACs on TNF-α-induced ICAM-1, VEGF and IL-8 expression in LT2 cells was then investigated. Basal ICAM-1, IL-8, VEGF_121 _and VEGF_165 _expression levels were low in unstimulated cells (Figure 6). Treatment of cells with TNF-α for 12 h significantly increased the expression of ICAM-1, IL-8, VEGF_121 _and VEGF_165_. Treatment of cells with PACs alone had no effect on the basal expression of ICAM-1, VEGF and IL-8 genes by LT2 cells (data not shown). A significant inhibition in inducible ICAM-1 expression was observed in a dose-dependent way in TNF-α stimulated cells (Figure 6A). PACs did not significantly modify IL-8 (Figure 6B) nor VEGF_165 _(Figure 6D) expression in TNF-α stimulated cells while it increased dose-dependently the expression of VEGF_121 _(Figure 6C).

The downregulation effects of PACs on inducible ICAM-1 expression were not a result of cytotoxicity, because no significant difference of viability was observed between non-treated or PACs-treated cells (up to 100 μg/ml) using the MTT assay.

## Discussion

Our results confirm and extend previous data [14-16,18] showing that proanthocyanidins (PACs) from *Ribes nigrum *leaves reduce carrageenin-induced pleurisy. The volume of the exudate was dose-dependently diminished by PACs. In this fluid, PACs decreased IL-1β, TNF-α, CINC-1 and nitrite/nitrate levels [18]. Simultaneously, the levels of leukocytes, mostly polymorphonuclear cells, were decreased. The transvasation of leukocytes from blood to the inflammatory exudate requires activation of leukocytes and expression of adhesion molecules on the surface of endothelial cells. We have examined both processes. PACs did not significantly reduce the expression of adhesion molecules on the surface of leukocytes but greatly reduced the production of adhesion molecules on the surface of endothelial cells *in vivo *during carrageenin-induced pleurisy.

Three adhesion molecules expressed by leukocytes were examined. LFA-1 (CD11a/CD18) is not stored within the leukocytes, its upregulation most likely results from a conformational change that allows the leukocyte to interact with ICAM-1 [3]. All the animals treated with saline or carrageenin in the pleural cavity or PACs showed the same level of LFA-1 expression. This lack of difference could depend on the fact that our antibody cannot differentiate quiescent LFA-1 from activated LFA-1. On the other hand, PACs did not reduce Mac-1, an inducible β2-integrin, but increased its expression at high doses. While low doses of PACs reduced VLA-4 expression, higher doses of PACs increased its mobilization to the membrane. The influence of PACs on adhesion molecules on leukocytes was thus variable and cannot explain the reduction of leukocyte accumulation in the pleural fluid.

In pulmonary tissue, our immunohistochemical studies showed a dose-dependent decrease in the expression of VCAM-1 and ICAM-1. These results were confirmed by the examination of ICAM-1 production by LT2 endothelial cells *in vitro*. PACs dose-dependently reduced ICAM-1 mRNA production, suggesting that PACs inhibited ICAM-1 production at the mRNA level. Previously, it was shown that other compounds of the proanthocyanidin family inhibit the expression of endothelial adhesion molecules, in systemic sclerosis patients [21], in keratinocytes [22] and in HUVEC [23]. In LT2 endothelial cells, the level of ICAM-1 mRNA was increased 25-fold by TNF-α. This overproduction would depend on the activation of NF-κB or JNK/AP-1 transduction pathway. Previous results from our laboratory (data not shown) have revealed that PACs are able to inhibit NF-κB activation but at concentrations 10 times higher than the dose used to inhibit ICAM-1 mRNA expression in LT2 cells. On the other hand, compounds from the proanthocyanidin family have been suggested to inhibit the activation of JNK-1 and c-JUN [23]. Thus the inhibition of the expression of adhesion molecules by PACs could be related to an interference of JNK/c-JUN/AP-1 pathway activation.

The inhibition of ICAM-1 mRNA production by PACs was rather specific as PACs did not reduce IL-8 mRNA and VEGF mRNA in LT2 cells. Although, we have observed that *in vivo *PACs inhibited CINC-1 production during carrageenin-induced pleurisy, these compounds did not affect the production of similar molecules such as IL-8 by LT2 endothelial cells. On the other hand, PACs interact with VEGF mRNA expression *in vitro*. VEGF functions as an endothelial growth and survival factors and also increases vascular permeability [5]. It may play a relevant role in neutrophil migration. Five human VEGF mRNA species encoding VEGF isoforms of, among them, the most abundant 121 and 165 amino acids are produced by alternative splicing of VEGF mRNA [24]. The deletion in VEGF_121 _begins at codon 116 of the mature form of VEGF_165 _[24]. PACs did not influence expression of VEGF_165 _mRNA but provoked an increase in VEGF_121 _expression. VEGF production is stimulated by cytokines such as IL-6 [25], IL-1β [26] and TNF-α [27]. Up-regulation of inducible VEGF in human HaCaT keratinocytes by compounds from the proanthocyanidin family was also observed by Khanna et al. [28,29], who correlated this property to redox activity of these products. Proanthocyanidins are, in fact, known to display anti-oxidant and free radical scavenging properties [30-34]. This effect of VEGF up-regulation may have a beneficial role in regulating and facilitating wound healing.

In conclusion, our current study provides evidence to support that the anti-inflammatory activity displayed by proanthocyanidins from blackcurrant leaves is related to down-regulation of endothelial adhesion molecules, ICAM-1 and VCAM-1, probably through the transcription factor AP-1 regulation pathway. It would be of great interest in the near future to determine the effect of PACs and of similar compounds on the JNK/c-Jun/AP-1 regulatory pathway. Moreover, PACs are capable of modulating inducible VEGF transcription. The role of PACs in wound healing still remains to be determined.

## Competing interests

The author(s) declare that they have no competing interests.

## Authors' contributions

NG carried out PACs isolation, immunohistochemistry, cell culture, RT-PCR, statistical analysis, participated in animal experimentation, and drafted the manuscript. MK carried out flow cytometry and participated in animal experimentation. BN coordinated cell culture and RT-PCR techniques. DD coordinated and helped in immunohistochemistry. JD participated in animal experimentation, in the concept, design and coordination of the study and helped to draft the manuscript. All authors read and approved the final manuscript.

## References

[B1] Carlos TM, Harlan JM (1994). Leukocyte-endothelial adhesion molecules. Blood.

[B2] Panés J, Perry M, Granger DN (1999). Leukocyte-endothelial cell adhesion: avenues for therapeutic intervention. Br J Pharmacol.

[B3] Panés J, Granger DN (1998). Leukocyte-endothelial cell interactions: molecular mechanisms and implications in gastro-intestinal disease. Gastroenterology.

[B4] Elices MJ, Osborn L, Takada Y, Crouse C, Luhowskyj S, Hemler M, Lobb RR (1990). VCAM-1 on activated endothelium interacts with the leukocyte integrin VLA-4 at a site distinct from the VLA-4/fibronectin binding site. Cell.

[B5] Neufeld G, Cohen T, Gengrinovitch S, Poltorak Z (1999). Vascular endothelial growth factor (VEGF) and its receptors. FASEB J.

[B6] Baggiolini M, Dewald B, Moser B (1994). Interleukin-8 and related chemotactic cytokines-CXC CC chemokines. Adv Immunol.

[B7] Burns AR, Smith CW, Walker DC (2003). Unique structural features that influence neutrophil emigration into the lung. Physiol Rev.

[B8] Hood JD, Meininger CJ, Ziche M, Granger HJ (1998). VEGF upregulates ecNOS message, protein, and NO production in human endothelial cells. Am J Physiol.

[B9] Gaudry M, Bregerie O, Andrieu V, El Benna J, Pocidalo MA, Hakim J (1997). Intracellular pool of vascular endothelial growth factor in human neutrophils. Blood.

[B10] Taichman NS, Young S, Cruchley AT, Taylor P, Paleolog E (1997). Human neutrophils secrete vascular endothelial growth factor. J Leukoc Biol.

[B11] Seo SM, McIntire LV, Smith CW (2001). Effects of IL-8, Gro-alpha, and LTB4 on the adhesive kinetics of LFA-1 and Mac-1 on human neutrophils. Am J Physiol Cell Physiol.

[B12] Tits M, Angenot L, Poukens P, Warin R, Dierckxsens Y (1992). Prodelphinidins from *Ribes nigrum*. Phytochemistry.

[B13] Tits M, Poukens P, Angenot L, Diercksens Y (1992). Thin-layer chromatographic analysis of proanthocyanidins from *Ribes nigrum *leaves. J Pharm Biomed Anal.

[B14] Tits M, Angenot L, Damas J, Dierckxsens Y, Poukens P (1991). Anti-inflammatory prodelphinidins from black currant (*Ribes nigrum*) leaves. Planta Med.

[B15] Garbacki N, Angenot L, Bassleer C, Damas J, Tits M (2002). Effects of prodelphinidins isolated from *Ribes nigrum *on chondrocytes metabolism and COX activity. Naunyn-Schmiedeberg's Arch Pharmacol.

[B16] Garbacki N, Tits M, Damas J (2003). Anti-inflammatory effect of natural proanthocyanidins : pharmacological evaluation on in vivo models. Pflügers Archiv Eur J Physiol.

[B17] Garbacki N, Damas J (2004). Some effects of proanthocyanidins isolated from *Ribes nigrum *on the cardiovascular system of the rat. Fund Clin Pharmacol.

[B18] Garbacki N, Tits M, Angenot L, Damas J (2004). Inhibitory effects of proanthocyanidins from *Ribes nigrum *leaves on carrageenin acute inflammatory reactions induced in rats. BMC Pharmacol.

[B19] Mosmann T (1983). Rapid colorimetric assay for cellular growth and survival: application to proliferation and cytotoxicity assays. J Immunol Meth.

[B20] Deroanne CF, Bonjean K, Servotte S, Devy L, Colige A, Clausse N, Blacher S, Verdin E, Foidart JM, Nusgens BV, Castronovo V (2002). Histone deacetylases inhibitors as anti-angiogenic agents altering vascular endothelial growth factor signaling. Oncogene.

[B21] Kalfin R, Righi A, Del Rosso A, Bagchi D, Generini S, Matucci Cerinic M, Das DK (2002). Activin, a grape seed-derived proanthocyanidin extract, reduces plasma levels of oxidative stress and adhesion molecules (ICAM-1, VCAM-1, and E-selectin) in systemic sclerosis. Free Rad Res.

[B22] Bito T, Roy S, Sen CK, Packer L (2000). Pine bark extract pycnogenol downregulates IFN-γ-induced adhesion of T cells to human keratinocytes by inhibiting inducible ICAM-1 expression. Free Rad Biol Med.

[B23] Bagchi D, Sen CK, Ray SD, Das DK, Bagchi M, Preuss HG, Vinson JA (2003). Molecular mechanisms of cardioprotection by a novel grape seed proanthocyanidin extract. Mut Res.

[B24] Houck KA, Ferrara N, Winer J, Cachianes G, Li B, Leung DW (1991). The vascular endothelial growth factor family-identification of a fourth molecular species and characterization of alternative splicing of RNA. Mol Endocrinol.

[B25] Cohen T, Nahari D, Cerem-Weiss L, Neufeld G, Levi B (1996). Interleukin-6 induces the expression of vascular endothelial growth factor. J Biol Chem.

[B26] Li J, Perrella MA, Tsai JC, Yet SF, Hsieh CM, Yoshizumi M, Patterson C, Endege WO, Zhou F, Lee ME (1995). Induction of vascular endothelial growth factor gene expression by interleukin-1 beta in rat aortic smooth muscle cells. J Biol Chem.

[B27] Ryuto M, Ono M, Izumi H, Yoshida S, Weich HA, Kohno K, Kuwano M (1996). Induction of vascular endothelial growth factor by tumor necrosis facto alpha in human glioma cells – possible role of SP-1. J Biol Chem.

[B28] Khanna S, Roy S, Bagchi D, Bagchi M, Sen CK (2001). Upregulation of oxidant-induced VEGF expression in cultured keratinocytes by a grape seed proanthocyanidin extract. Free Rad Biol Med.

[B29] Khanna S, Venojarvi M, Roy S, Sharma N, Trikha P, Bagchi D, Bagchi M, Sen CK (2002). Dermal wound healing properties of redox-active grape seed proanthocyanidins. Free Rad Biol Med.

[B30] Maffei Facino R, Carini M, Aldini G, Berti F, Rossoni G, Bombardelli E, Morazzoni P (1996). Procyanidins from *Vitis vinifera *seeds protect rabbit heart from ischemia/reperfusion injury: antioxidant intervention and/or iron and copper sequestering ability. Planta Med.

[B31] Aucamp J, Gaspar A, Hara Y, Apostolides Z (1997). Inhibition of xanthine oxidase by catechins from tea (*Camellia sinensis*). Anticancer Res.

[B32] Bagchi D, Garg A, Krohn RL, Bagchi M, Bagchi DJ, Balmoori J, Stohs SJ (1998). Protective effects of grape seed proanthocyanidins and selected antioxidants against TPA-induced hepatic and brain lipid peroxidation and DNA fragmentation and peritoneal macrophage activation in mice. Gen Pharmacol.

[B33] Zhao J, Wang J, Chen Y, Agarwal R (1999). Anti-tumor-promoting activity of a polyphenolic fraction isolated from grape seeds in the mouse skin two-stage initiation-promotion protocol and identification of procyanidin B5-3'-gallate as the most effective antioxidant constituent. Carcinogenesis.

[B34] Bagchi D, Bagchi M, Stohs SD, Ray SD, Sen CK, Preuss HG (2002). Cellular protection with proanthocyanidins derived from grape seeds. Ann NY Acad Sci.

